# ATF7‐dependent epigenetic changes induced by high temperature during early porcine embryonic development

**DOI:** 10.1111/cpr.13352

**Published:** 2022-10-18

**Authors:** Ming‐Hong Sun, Wen‐Jie Jiang, Xiao‐Han Li, Song‐Hee Lee, Geun Heo, Dongjie Zhou, Jung‐Seok Choi, Kwan‐Suk Kim, Wenfa Lv, Xiang‐Shun Cui

**Affiliations:** ^1^ Department of Animal Science Chungbuk National University Cheongju South Korea; ^2^ College of Animal Science and Technology Jilin Agricultural University Changchun China

## Abstract

**Background:**

Activating transcription factor 7 (ATF7) is a member of the ATF/cAMP response element (CRE) B superfamily. ATF2, ATF7, and CRE‐BPa are present in vertebrates. *Drosophila* and fission yeast have only one homologue: dATF2 and Atf1, respectively. Under normal conditions, ATF7 promotes heterochromatin formation by recruiting histone H3K9 di‐ and tri‐methyltransferases. Once the situation changes, all members are phosphorylated by the stress‐activated kinase P38 in response to various stressors. However, the role of ATF7 in early porcine embryonic development remains unclear.

**Results:**

In this study, we found that ATF7 gradually accumulated in the nucleus and then localized on the pericentric heterochromatin after the late 4‐cell stage, while being co‐localized with heterochromatin protein 1 (HP1). Knockdown of ATF7 resulted in decreases in the blastocyst rate and blastocyst cell number. ATF7 depletion resulted in downregulation of HP1 and histone 3 lysine 9 dimethylation (H3K9me2) expression. These effects were alleviated when P38 activity was inhibited. High temperatures increased the expression level of pP38, while reducing the quality of porcine embryos, and led to ATF7 phosphorylation. The expression level of H3K9me2 and HP1 was decreased and regulated by P38 activity.

**Conclusion:**

Stress‐induced ATF7‐dependent epigenetic changes play important roles in early porcine embryonic development.

## Graphical abstract

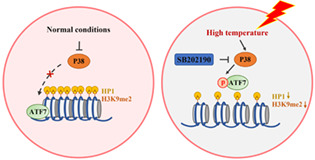

## 1 BACKGROUND

There are two types of chromatin in eukaryotic genomes, characterized according to its structure and compaction state: euchromatin and heterochromatin.^1^, ^2^ Euchromatin is found in endosomes in the nucleus and has an open, unfolded structure that allows the transcription machinery to bind to DNA, thereby facilitating transcription.^3^ The majority of chromatin is present in the form of heterochromatin, which is spatially separated from euchromatin within the nucleus and preferentially localizes to the nuclear periphery and surrounds the nucleolus. Functions of heterochromatin range from silencing of gene expression to restricting DNA replication to DNA repair.^4^ Heterochromatin includes regions of the genome that can be densely stained with chemical dyes. These regions are rich in repetitive elements and epigenetic markers, including histone H3 lysine 9 dimethylation/trimethylation (H3K9me2/H3K9me3) and histone H3 lysine 27 trimethylation (H3K27me3).^5^ A distinct feature during oogenesis in some mammalian species is the formation of heterochromatin rings called the karyosphere, which are associated with the periphery of nucleolus‐like bodies. The acquisition of specific morphological structures through heterochromatin is also characteristic of zygotes.^6^ Studies on bovine,^7^ porcine,^8^ and human^9^ embryos have shown that the spatial arrangement of specific heterochromatin regions in the nuclear periphery is a common feature of mammalian cleavage initiation stages.

At the time of zygotic genome activation (ZGA) during early embryonic development, large domains of active or inactive chromatin often cluster together to form euchromatic A‐ or heterochromatic B‐compartments, respectively. At this stage, HP1 plays an important role by establishing the embryonic heterochromatin structure, which appears to mediate the clustering and condensation of constitutive heterochromatin at pericentromeric regions through H3K9me2/3‐dependent binding, the overall configuration of the auxiliary chromosome arms, and contribut to the formation of the B‐compartment.^10^ Iovino, and Giorgetti have proposed that loss of HP1 from heterochromatin causes decreased interactions between B‐compartment regions, resulting in declustering and decondensation and leading to reduced segregation of B‐compartments.^11^ In addition, in the ovaries of postnatal female mice, growing oocytes gradually accumulate H3K9me2,^12^ and after fertilization, zygotes exhibit an asymmetric pattern of H3K9me2,^13^ the histone H3 lysine 9 dimethylation (H3K9me2) is mediated by the methyltransferase G9a, which is essential for embryonic development. Previous studies have shown that deletion of G9a in embryos results in lack of substantial accumulation of H3K9me2, resulting in slight developmental delay and destabilization of the ICM lineage, as well as frequent embryo loss during the preimplantation stage.^14^ Therefore, HP1 and H3K9me2 are essential for embryonic development.

Activating transcription factor 7 (ATF7) is a vertebrate member of the ATF2 subfamily belonging to the ATF/cAMP response element (CRE) B (CREB) superfamily.^15^, ^16^, ^17^ In addition to ATF7, it contains two members of ATF2 and CRE‐BPa in vertebrates. *Drosophila* and fission yeast have only one homologue (dATF2 and Atf1, respectively). All three members contain a B‐ZIP‐type DNA‐binding domain and a trans‐activation domain consisting of a metal finger structure and stress‐activated protein kinase (SAPK) phosphorylation sites, such as P38 and Jun N‐terminal protein kinase (JNK), which are expressed in various tissues and cells.^18^ SAPKs are activated by various stresses, such as metabolism‐related reactive oxygen species (ROS), and environmental stresses, such as hypoxia, osmotic stress, and heat stress.^19^, ^20^ ATF2 is phosphorylated by both P38 and JNK, while only P38 has been shown to phosphorylate ATF7, but not other kinase(s).^21^ In the absence of stress, ATF7 plays a role in gene silencing through the formation of heterochromatin‐like structures. In various types of somatic cells, vertebrate ATF7 silences its target genes by recruiting H3K9 di‐ and tri‐methyltransferases, G9a, Suv39h1, and ERG‐associated protein with SET domain/SET domain bifurcated histone lysine methyltransferase 1 (ESET/SETDB1).^22^, ^23^ ATF7 represses a group of innate immunity‐related genes in macrophages by associating with H3K9 di‐methyltransferase G9a. Studies also showed that Atf1, dATF2 and ATF7 contribute to heterochromatin formation by recruiting histone H3K9 di‐methyltransferases independently from the RNA interference (RNAi)‐dependent mechanism.^24^ In response to various stresses, ATF7 is phosphorylated by P38, which leads to the release of ATF7 and H3K9 di‐ and tri‐methyltransferases, ultimately reducing H3K9me2 or H3K9me3 levels and disrupting heterochromatin‐like structures, which is a long‐lasting injury.^25^ Pathogen‐infection‐induced phosphorylation of ATF7 stimulates the release of ATF7‐G9a from target genes, accompanied by a decrease in repressive histone H3K9me2 levels, leading to elevated gene expression. Also, in macrophages, ATF7 is phosphorylated by P38 and released from chromatin following lipopolysaccharide treatment, leading to disruption of heterochromatin and a reduction in the level of H3K9me2. Once ATF7 is released from chromatin, it is not entirely recruited back to the original binding sites.^26^

As an important factor of environmental exposure, heat stress has attracted attention for its effects on embryonic development *in vivo* and *in vitro* in pigs^27^, ^28^ and cattle.^29^, ^30^ ATF7 plays a key role as an epigenetic regulator of stress responses during environmental stress‐induced heterochromatin disruption. However, the role of ATF7 in heat‐induced chromatin changes during early porcine embryonic development has not been investigated.

In the present study, we reduced the expression of ATF7 by microinjection of ATF7 double‐strand RNA (dsRNA) to explore the role of ATF7 during early porcine embryonic development. In addition, embryos were treated at high temperatures to explore the role of ATF7 in stress response. Our results suggest that ATF7 contributes to heterochromatin and that heat‐induced pATF7 activation disrupts heterochromatin, which leads to the failure of blastocyst formation during early porcine embryonic development.

## 2 METHODS

All chemicals were purchased from Millipore Sigma (Burlington, MA, USA) unless otherwise indicated. All manipulations were performed on a heated stage adjusted to 38.5°C unless otherwise indicated.

### 2.1 Ethics statement

This study was performed in accordance with the guidelines of the Institutional Animal Care and Use Committee of the Laboratory Animal Center of Chungbuk National University, Cheongju, South Korea. All operations related to the mice were performed according to the guidelines of the committee.

### 2.2 Collection and *in vitro* maturation of porcine oocytes

The ovaries of prepubertal gilts collected at a local slaughterhouse were placed in 37°C saline containing 75 mg/ml penicillin G and 50 mg/ml streptomycin sulphate. The ovaries were transported to the laboratory within 3 h. Cumulus–oocyte complexes (COCs) were aspirated from 3 to 6 mm ovarian follicles using a 10 ml disposable syringe attached to an 18‐gauge needle. Oocytes with at least three layers of compact cumulus cells and a uniform ooplasm were selected for *in vitro* maturation (IVM). After supplementation with 0.1 g/L sodium pyruvate, 0.6 mmol/L l‐cysteine, 10 ng/ml epidermal growth factor, 10% (v/v) porcine follicular fluid, 10% (v/v) porcine follicular fluid, 10 IU/ml luteinizing hormone, and 10 IU/ml follicle‐stimulating hormone in the *in vitro* maturation medium (TCM‐199, 11150‐059; Gibco, Franklin Lakes, NJ, USA), 500 μl of maturation medium per well was added to a 4‐well dish and covered with 300 μl of mineral oil. Up to 120 COCs that had been washed three times were transferred to each well and incubated for 44 h at 38.5°C in a humidified atmosphere containing 5% CO_2_.

### 2.3 Production of parthenogenetic activation embryos and *in vitro* culture

COCs were placed in 1 mg/ml hyaluronidase to remove cumulus cells by pipetting approximately 50 times. MII‐stage denuded oocytes were selected. They were electrically activated by two direct‐current pulses of 120 V for 60 μs in 297 mmol/L mannitol (pH 7.2) containing 0.1 mmol/L CaCl_2_, 0.05 mmol/L MgSO_4_, 0.01% (w/v) polyvinyl alcohol (PVA), and 0.5 mmol/L HEPES. These oocytes were then cultured in bicarbonate‐buffered porcine zygote medium 5 (PZM‐5) containing 7.5 μg/ml cytochalasin B and 4 mg/ml bovine serum albumin (BSA) for 3 h to suppress extrusion of pseudo‐second polar bodies according to the manufacturer's instruction.^31^ The activated oocytes were thoroughly washed and divided into five groups: (a) control group, (b) ATF7‐knockdown (ATF7‐KD) group, (c) ATF7‐KD group with SB202190 inhibitor (ATF7‐KD + SB202190), (d) high temperature group (HT), and (e) HT group with SB202190 inhibitor (HT + SB202190). The different groups were cultured in bicarbonate‐buffered PZM‐5 supplemented with 4 mg/ml BSA in 4‐well plates for 7 days at 38.5°C in a humidified atmosphere containing 5% CO_2_. The HT and HT + SB202190 groups were cultured at 39.5°C or 40.5°C.

### 2.4 Inhibitor preparation and treatment

SB202190, P38 MAPK inhibitor, was prepared as a 10 mM stock and serially diluted to 0, 0.1, and 0.5 μM working concentrations. To investigate whether P38 activity mediates the effect of HT on porcine early embryos, we supplemented IVC with different concentrations of SB202190 at the same time as HT treatment of activated oocytes. Embryos were cultured to the 4‐cell stage, washed three times, and placed in fresh IVC for subsequent culture.

### 2.5 Preparation and injection of *ATF7* dsRNA preparation and microinjection

To prepare *ATF7* dsRNA, *ATF7* was amplified using a pair of primers containing the T7 promoter sequence (*ATF7* dsRNA: forward, TAA TAC GAC TCA CTA TAG GGG GGC TAT GAT CCA CTT CAC CC; reverse, TAA TAC GAC TCA CTA TAG GGG CTC ATC CGG ATC TTC ATC CA). The purified PCR products were used to synthesize dsRNA using the MEGAscript T7 Kit (AM1333; Thermo Fisher Scientific, Waltham, MA, USA) according to the manufacturer's instructions.^32^, ^33^ After *in vitro* transcription, dsRNA was treated with DNase I and RNase A to remove the DNA template and any single‐stranded RNA, followed by purification by phenol–chloroform extraction and isopropyl alcohol precipitation. The purified dsRNA was dissolved in RNase‐free water. The concentration was determined by measuring the optical density at 260 nm (Nanodrop, Thermofisher, Deutsch, Germany) and adjusted to a final concentration of 1 μg/μl dsRNA aliquots was stored at −80°C until use.

For knockdown experiments, *ATF7* dsRNA was microinjected into the cytoplasm of an oocyte following parthenogenetic activation (PA) and 3 h of CB treatment using a Femto‐Jet electronic microinjector (Eppendorf AG, Hamburg, Germany) and a Diaphot ECLIPSE TE300 inverted microscope (Nikon, Tokyo, Japan) equipped with a model MM0‐202N hydraulic three‐dimensional micromanipulator (Narishige Inc., Tokyo, Japan). After injection, the embryos were cultured in PZM‐5.

### 2.6 Immunofluorescent staining and confocal microscopy

The embryos in the control, ATF7‐KD, HT, ATF7‐KD + SB202190, and HT + SB202190 groups were fixed for 1 h with 3.7% paraformaldehyde at room temperature. After permeabilization in 0.5% Triton X‐100 (in PBS/PVA) for 1 h at room temperature, embryos were transferred to blocking buffer (1% BSA in PBS) for 1 h at room temperature or overnight at 4°C. For ATF7 staining, embryos were incubated with rabbit anti‐ATF7 polyclonal antibody (LS‐C354157, 1:100 dilution; LSBIO, Seattle, WA, USA) overnight at 4°C and after washing with PBS/PVA three times for 3 min each time, embryos were incubated with Alexa Fluor 546‐conjugated goat anti‐rabbit IgG (1:200) for 1 h at room temperature. To visualize heterochromatin protein 1 (HP1), embryos were incubated with Immunotag™ HP1 monoclonal antibody‐488 (1:100 dilution; ImmunoTag, Noida, India) overnight at 4°C. After washing three times in the same manner, embryos were incubated with Hoechst 33342 at room temperature for 15 min. Finally, the embryos were mounted on glass slides and observed by confocal laser‐scanning microscopy using a model LSM 710 META microscope (Carl Zeiss, Jena, Germany).

### 2.7 Real‐time quantitative PCR

Embryos were collected and mRNA was extracted from a pool of 30 embryos per group using the DynaBeads mRNA Direct Kit (61,012; Thermo Fisher Scientific) according to the manufacturer's instructions.^34^ cDNA was obtained by reverse transcription of mRNA using the Oligo(deoxythymine) 20 primer and SuperScript III Reverse Transcriptase (Thermo Fisher Scientific). Amplification was performed at 95°C for 3 min, followed by 40 cycles of 95°C for 15 s, 60°C for 25 s, 72°C for 10 s, and final extension at 72°C for 5 min. The target gene and housekeeping were *ATF7* (forward, AAA GCC CCA AGG AAA GCT CA; reverse, AGA CTG AGA CTG TGG GGT CA)，*G9a* (forward, GAA GGT GAC CTC AGA CGT GG; reverse, CCA CTC GCT CAT CCA CAG AG), *SUV39H2* (forward, ATT CCA CCA GGT ACC CCC AT; reverse, CCA CAG CCA TTG CTA GTT CG) and *18s* (forward, CGC GGT TCT ATT TTG TTG GT; reverse, AGT CGG CAT CGT TTA TGG TC), respectively. The mRNA quantification data were analysed using the 2^−ΔΔCt^ method.

### 2.8 Western blotting

We lysed at least 70 embryos from different groups with 20 μl of ice‐cold Laemmli sample buffer (sodium dodecyl sulphate [SDS] sample buffer containing 2‐mercaptoethanol). The buffer was heated at 95°C for 10 min and stored at −20°C. According to previous article,^35^ proteins in each sample were separated by 10% SDS‐polyacrylamide gel electrophoresis (SDS‐PAGE) and transferred to a polyvinylidene fluoride (PVDF) membrane (Millipore, Bedford, MA, USA) via electroblotting. Non‐specific binding sites were blocked with Tris‐buffered saline containing Tween‐20 (TBST) and either 5% skimmed milk powder or BSA for 1 h at room temperature. The membranes were then incubated with mouse anti‐ATF7 polyclonal antibody (1:1000) in blocking solution overnight at 4°C. After washing in TBST three times (10 min each), the membranes were incubated with a horseradish peroxidase (HRP)‐conjugated secondary antibody (1:1000) for 1 h at room temperature. Finally, the membranes were exposed to SuperSignal West Femto Maximum Sensitivity Substrate (Thermo Fisher Scientific). The band intensity values were analysed using the ImageJ software.

### 2.9 Statistical analysis

At least three replicates were performed in all experiments. Results are expressed as the mean ± SEM. Statistical analysis was performed using GraphPad Prism software (version 5.0; GraphPad Inc., San Diego, CA, USA). Statistical comparisons were performed using independent‐sample *t*‐tests. Statistical significance was set at *p* < 0.05. Fluorescence pixel intensities were analysed using ImageJ software (version 1.50; National Institutes of Health, Bethesda, MD, USA).

## 3 RESULTS

### 3.1 Expression and localization of ATF7 during porcine embryonic development

**FIGURE 1:**
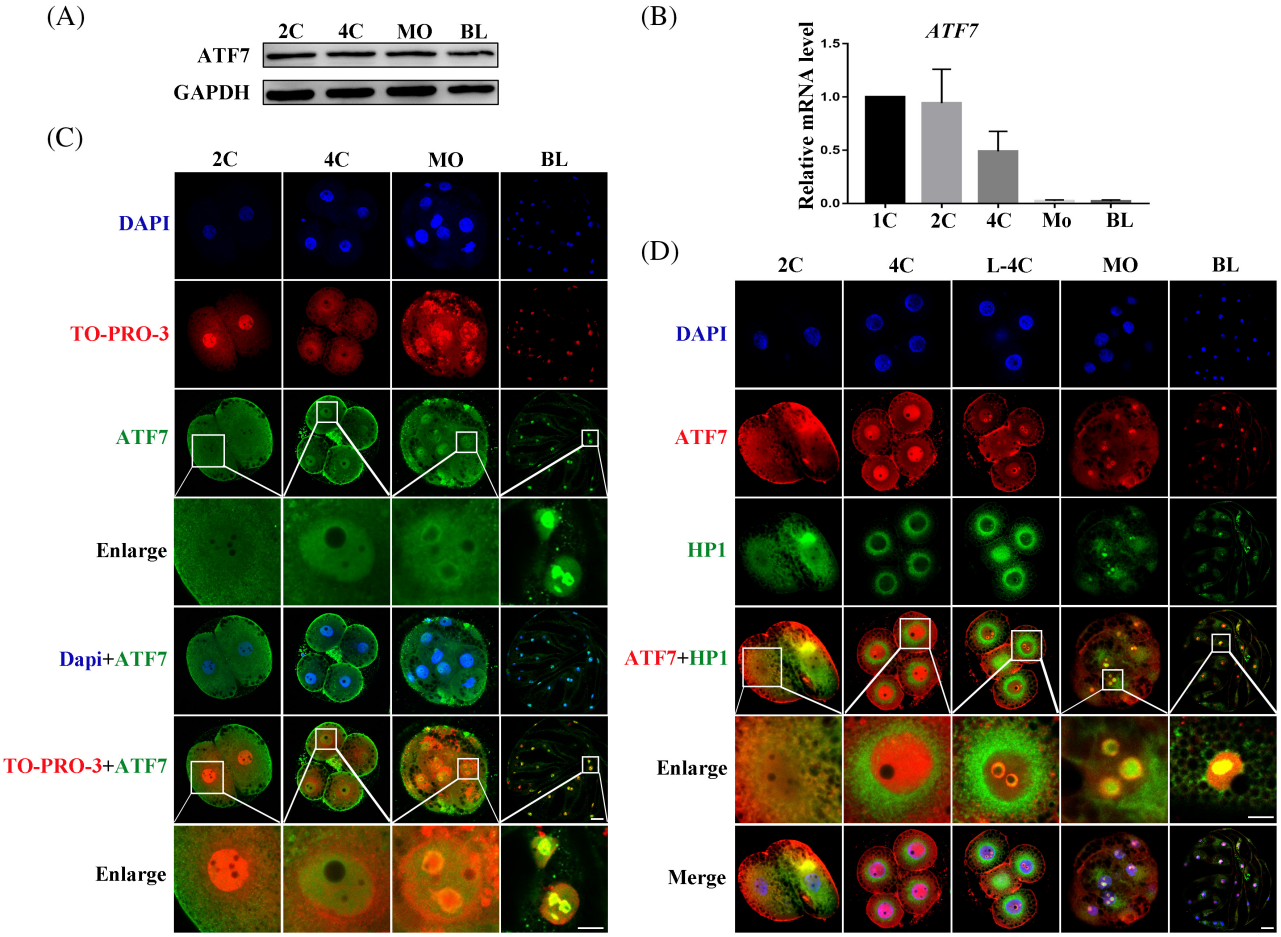
Expression and localization of ATF7 during porcine embryonic development. (A) Western blotting results of ATF7 protein expression levels during early porcine embryonic development. (B) Real‐time quantitative PCR results of *ATF7* mRNA expression levels during early porcine embryonic development. (C)ATF7 antibody to detect ATF7 localization during early porcine embryonic development. At the 2C stage, ATF7 was enriched near the nucleus. At the 4C stage, ATF7 entered the nucleus. When embryos were at the MO and BL stages, ATF7 was mainly localized on pericentric heterochromatin. Blue, DAPI; red, TO‐PRO‐3; green, ATF7; bar = 20 μm. (D) Typical picture of localization of ATF7 and HP1. Colocalization of ATF7 and HP1 was evident from the late 4‐cell to the BL stage. Blue, DAPI; red, ATF7; green, HP1; bar = 20 μm

To investigate the function of ATF7 in porcine early embryonic development, we first detected the expression of *ATF7* at all stages of embryonic development using western blotting and real‐time quantitative PCR. The embryos were cultured to the 2‐cell (2C), 4‐cell (4C), morula (MO), and blastocyst (BL) stages. As shown in Figure 1A, ATF7 was expressed at all embryo stages, the results of WB indicated that the protein expression level of ATF7 was similar and remained almost unchanged at various stages of embryonic development (2C, 1.00, *n* = 240; 4C, 1.05 ± 0.04, *n* = 240; MO, 1.08 ± 0.03, *n* = 240; BL, 1.02 ± 0.06, *n* = 240). In addition, as shown in Figure 1B, the mRNA expression level of *ATF7* decreased rapidly at 4C and was extremely low in the MO and BL stages (1C, 1.00, *n* = 120; 2C, 0.94 ± 0.18, *n* = 120; 4C, 0.49 ± 0.11, *n* = 120; MO, 0.02 ± 0.01, *n* = 120; BL, 0.02 ± 0.01, *n* = 120). Next, the embryos were stained with ATF7 antibody to examine their localization at the different stages. Embryo chromosomes were visualized by co‐staining with 4′,6‐diamidino‐2‐phenylindole (DAPI) and TO‐PRO‐3; bright regions corresponded to heterochromatin and dim areas to euchromatin. As shown in Figure 1C, ATF7 was enriched around the nucleus during the 2C stage. After cells entered 4C, ATF7 was localized in the nucleus and then accumulated in the pericentric heterochromatin in the nucleus until the BL stage. ATF7 co‐localized with HP1 on pericentric heterochromatin from the L‐4C (72 h after parthenogenesis) to BL stages (Figure 1D). The expression and localization of ATF7 in embryos suggest an important role for ATF7 in porcine early embryonic development.

### 3.2 Effects of ATF7 knockdown on early porcine embryonic development

**FIGURE 2:**
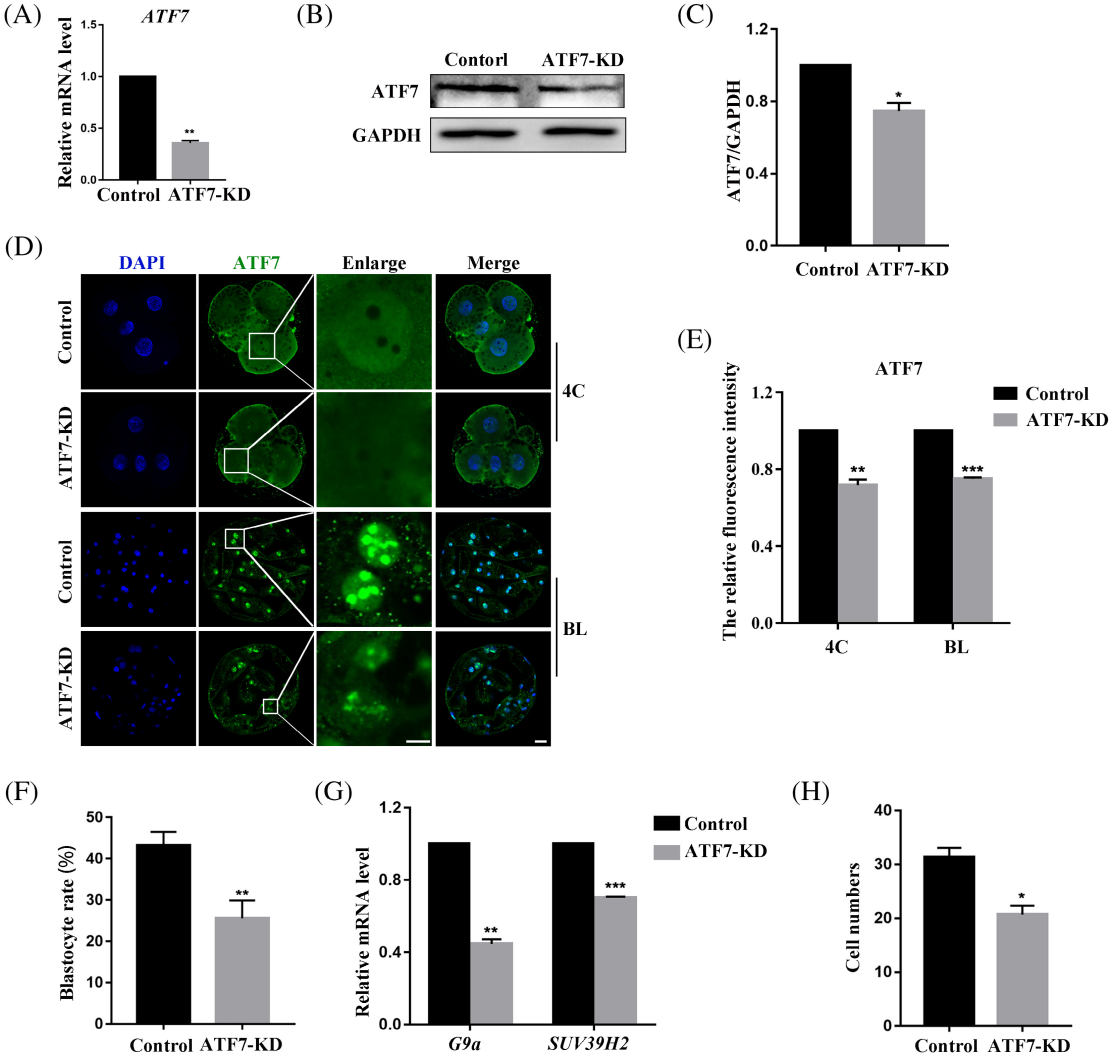
Effects of ATF7 knockdown on early porcine embryonic development. (A) Real‐time quantitative PCR results of *ATF7* mRNA expression levels in the control and ATF7‐KD groups. Compared with the control group, the expression level of *ATF7* mRNA was significantly lower in the ATF7‐KD group. **p < 0.01. (B) Western blotting results of ATF7 protein expression after microinjection of *ATF7* dsRNA. (C) Band intensity analysis of ATF7 after *ATF7* dsRNA microinjection. ATF7 protein expression decreased significantly in the ATF7‐KD group. *p < 0.05. (D) Typical pictures of ATF7 fluorescence signal at 4C and BL stages after *ATF7* dsRNA microinjection. Blue, DAPI; green, ATF7; bar = 20 μm. (E) Fluorescence intensity of ATF7 after *ATF7* dsRNA microinjection. Compared with the control group, ATF7 fluorescence intensities of 4C and BL in the ATF7‐KD group were significantly lower. **p < 0.01; ***p < 0.001. (F) Blastocyst rate after *ATF7* dsRNA microinjection. The rate reduced significantly in the ATF7‐KD group. **p < 0.01. (G) Real‐time quantitative PCR results of *G9a* and *SUV39H2* mRNA expression levels in the control and ATF7‐KD groups. Compared with the control group, the expression level of *G9a* and *SUV39H2* mRNA was significantly lower in the ATF7‐KD group. **p < 0.01; ***p < 0.001. (H) Cell numbers in blastocysts after *ATF7* dsRNA microinjection. The cell number of blastocysts was decreased in the ATF7‐KD group. *p < 0.05

To explore the potential function of ATF7 during early porcine embryonic development, the expression of ATF7 protein was knocked down by microinjection of dsRNA. Real‐time quantitative PCR and western blotting were used to examine the knockdown efficiency. After microinjection of *ATF7* dsRNA about 48 hours, the relative expression level of *ATF7* mRNA in the ATF7‐KD group of 4C embryos was significantly lower than that in the control group (control group, 1.00, *n* = 150; ATF7‐KD group, 0.36 ± 0.02, *n* = 150, *p* < 0.01) (Figure 2A). Western blotting revealed that ATF7 protein expression decreased significantly in the ATF7‐KD group, which was confirmed by densitometric analysis of the bands (control group, 1.00, *n* = 240; ATF7‐KD group, 0.75 ± 0.05, *n* = 240, *p* < 0.05) (Figure 2B,C). In addition, after ATF7 knockdown, ATF7 immunofluorescence staining revealed significantly decreased fluorescence intensity of ATF7 at the 4C and BL stages (4C: control group, 1.00, *n* = 30; ATF7‐KD group, 0.72 ± 0.03, *n* = 31, *p* < 0.01; BL: control group, 1.00, *n* = 28; ATF7‐KD group, 0.75 ± 0.01, *n* = 30, *p* < 0.001) (Figure 2D,E). Embryos at the 1C stage were cultured with knockdown of ATF7 expression *in vitro* for 7 days. The rate of blastocyst formation was significantly lower than that in the control group (control group, 43.20 ± 3.28%, *n* = 386; ATF7‐KD group, 25.59 ± 4.32%, *n* = 395, *p* < 0.01) (Figure 2F). As shown in Figure 2G, after ATF7 knockdown, the mRNA expression levels of H3K9me2 methyltransferase *G9a* and H3K9me3 methyltransferase *SUV39H2* were significantly decreased (*G9a*: control group, 1.00, *n* = 120; ATF7‐KD group, 0.45 ± 0.02, *n* = 120, *p* < 0.01; *SUV39H2*: control group, 1.00, *n* = 120; ATF7‐KD group, 0.70 ± 0.01, *n* = 120, *p* < 0.001). Moreover, the cell number of blastocysts was significantly reduced after ATF7 knockdown (control group, 31.33 ± 1.76, *n* = 31; ATF7‐KD group, 20.67 ± 1.67, *n* = 24, *p* < 0.05) (Figure 2H). These results suggest that ATF7 knockdown leads to decreased embryo quality.

### 3.3 Effects of ATF7 knockdown on heterochromatin in porcine embryos

**FIGURE 3:**
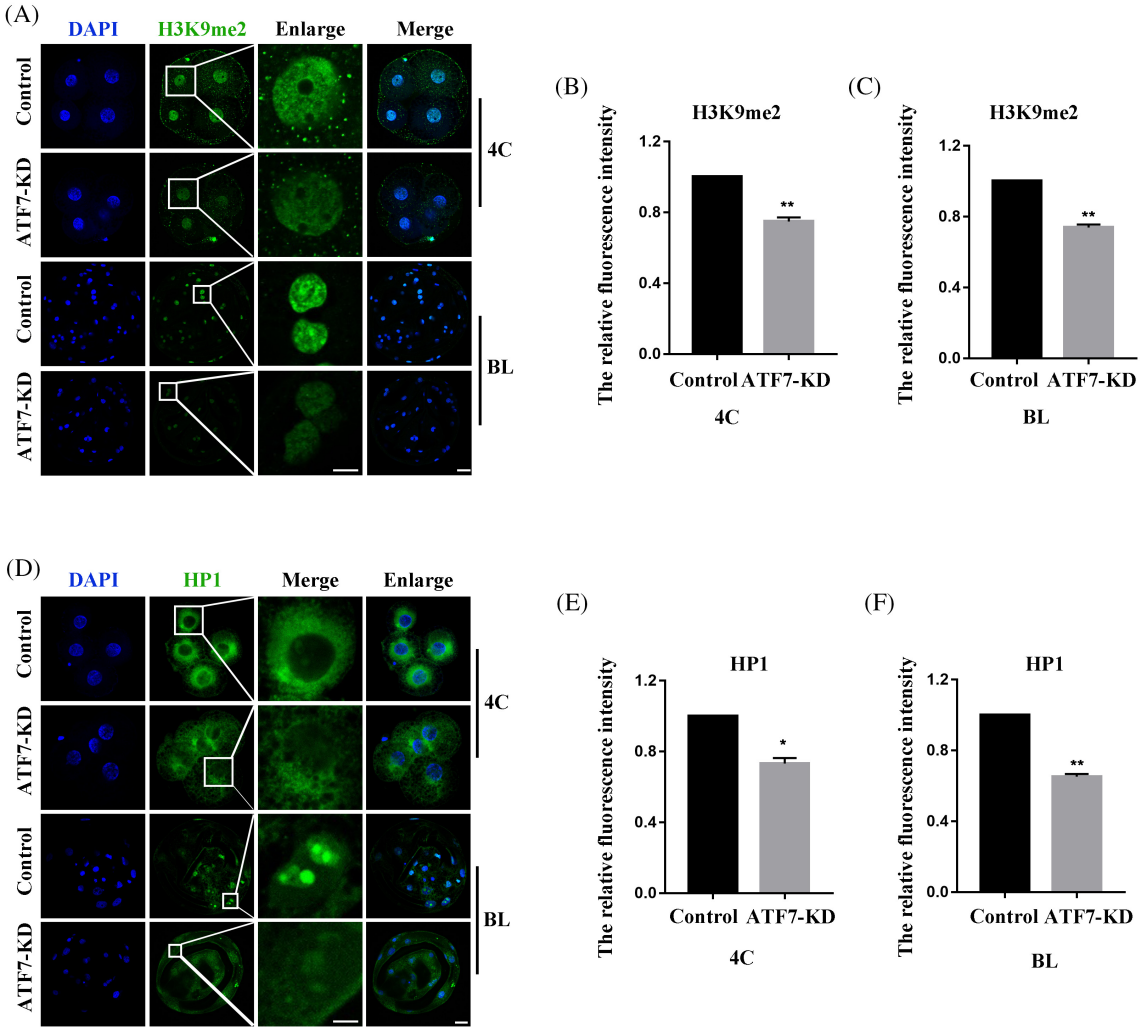
Effects of ATF7‐KD on heterochromatin in porcine embryos. (A) Typical pictures of H3K9me2 intensity in 4C and BL stages after *ATF7* dsRNA microinjection. Blue, DAPI; green, H3K9me2. (B,C) Fluorescence intensity of H3K9me2 in 4C and BL stages after ATF7 dsRNA microinjection. Compared with the control group, the relative fluorescence intensity of H3K9me2 in 4C and BL stages in the ATF7‐KD group was significantly lower. **p < 0.01. (D) Typical pictures of HP1 intensity in 4C and BL stages after *ATF7* dsRNA microinjection. Blue, DAPI; green, HP1; (E,F) fluorescence intensity of HP1 in 4C and BL stages after *ATF7* dsRNA microinjection. The relative fluorescence intensity of HP1 in 4C and BL stages in the ATF7‐KD group was significantly lower compared with the control group. *p < 0.05; **p < 0.01

Immunofluorescence staining revealed the co‐localization of ATF7 with HP1 from the L‐4C to BL stages. Since ATF7 has been shown to be essential for heterochromatin composition, we examined the effects of ATF7 on the heterochromatin marker protein HP1 and heterochromatin‐enriched histone H3K9me2. The expression levels of HP1 and H3K9me2 were determined after ATF7 knockdown by antibody staining. As shown in Figure 3A, the H3K9me2 protein signal in the 4C and BL stages was significantly decreased in the ATF7‐KD group compared with that in the control group. The fluorescence intensity analysis results were also consistent with this (4C: control group, 1.00, *n* = 30; ATF7‐KD group, 0.75 ± 0.02, *n* = 30, *p* < 0.01; BL: control group, 1.00, *n* = 36; ATF7‐KD group, 0.74 ± 0.02, *n* = 31, *p* < 0.01) (Figure 3B,C). In addition, the fluorescence intensity of HP1 was significantly decreased in 4C and BL in the ATF7‐KD group (4C: control group, 1.00, *n* = 45; ATF7‐KD group, 0.73 ± 0.03, *n* = 44, *p* < 0.05; BL: control group, 1.00, *n* = 60; ATF7‐KD group, 0.65 ± 0.02, *n* = 48, *p* < 0.01) (Figure 3D–F). Taken together, these data suggest that ATF7 is essential for heterochromatin function in porcine embryos.

### 3.4 Effects of inhibition of P38 on ATF7‐KD embryonic development

**FIGURE 4:**
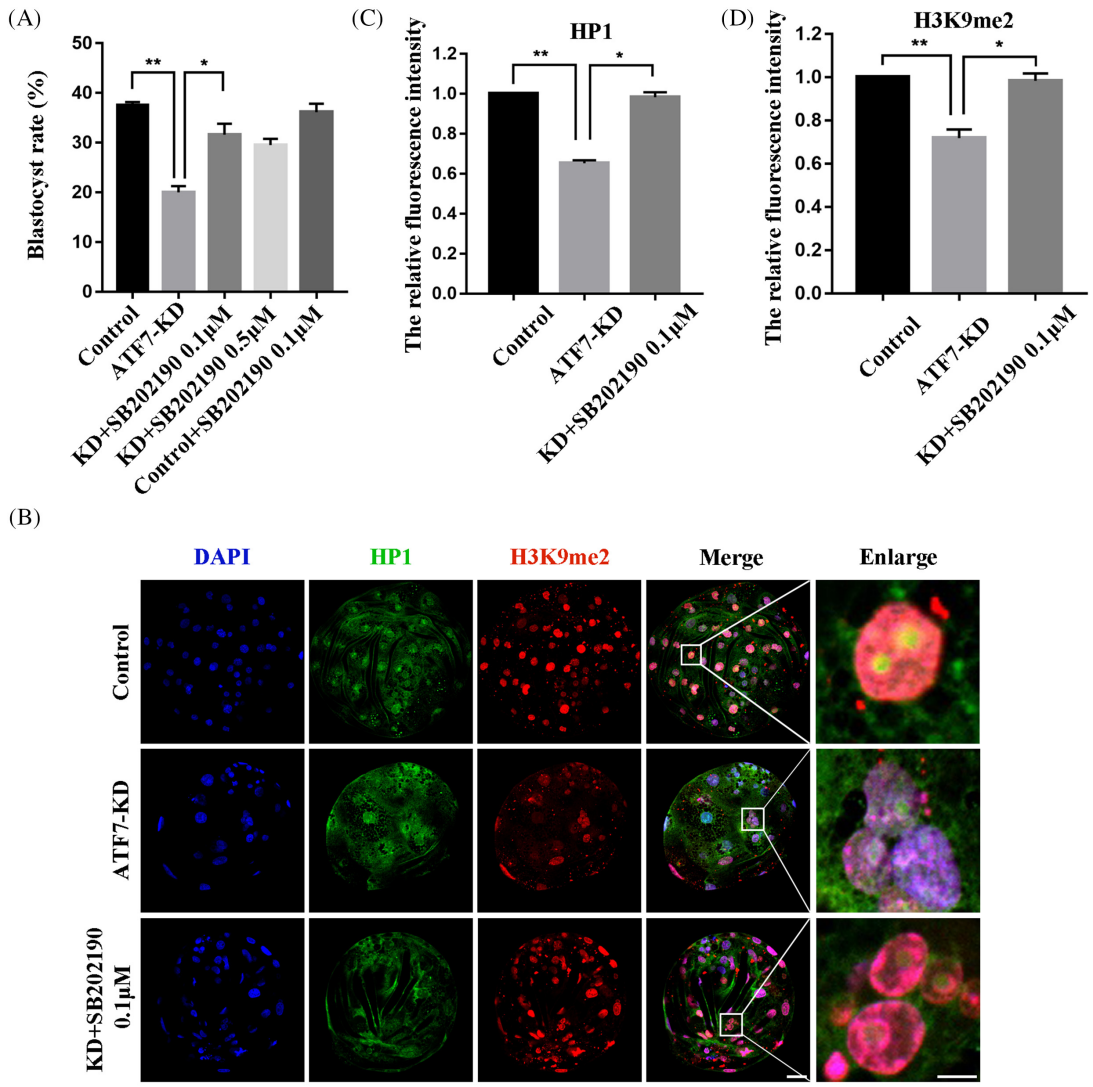
Effects of inhibition of P38 on ATF7‐KD embryonic development. (A) Blastocyst rate after *ATF7* dsRNA microinjection and treatment with SB202190 at 0.1 and 0.5 μM. The blastocyst rate was significantly lower in the ATF7‐KD group compared with the control group and was significantly higher in the ATF7‐KD + SB202190 0.1 μM group compared with the ATF7‐KD group. In addition, there was no significant difference in the control + SB202190 0.1 μM group compared to the control group. *p < 0.05; **p < 0.01. (B) Typical pictures of H3K9me2 and HP1 intensities in the BL stage after *ATF7* dsRNA microinjection and treatment with SB202190 at 0.1 μM. Blue, DAPI; green, HP1; red, H3K9me2. (C,D) Fluorescence intensity of HP1 and H3K9me2 in the BL stage after *ATF7* dsRNA microinjection. The relative fluorescence intensity of HP1 and H3K9me2 in the BL stage in the ATF7‐KD group were significantly lower compared with the control group. The relative fluorescence intensity of HP1 and H3K9me2 increased significantly in the ATF7‐KD + SB202190 0.1 μM group compared with the ATF7‐KD group. *p < 0.05; **p < 0.01

To explore whether background stress levels affect ATF7‐dependent heterochromatin function through P38, we further explored the effect of inhibiting P38 activity with the inhibitor SB202190 on early porcine embryos in the ATF7‐KD group. As shown in Figure 4A, the rate of blastocyst formation was significantly lower in the ATF7‐KD group than in the control group (control group, 37.59 ± 0.61%, *n* = 254; ATF7‐KD group, 20.04 ± 1.23%, *n* = 154, *p* < 0.01; control+SB202190 0.1 μM group, 36.14 ± 1.73%, *n* = 258, *p* > 0.05). Furthermore, blastocyst formation increased when embryos in the AT7‐KD group were cultured in IVC containing 0.1 μM or 0.5 μM SB202190. The rate of blastocyst formation in the AT7‐KD + 0.1 μM SB202190 group was significantly increased compared with the AT7‐KD group (ATF7‐KD + SB202190 0.1 μM group, 31.59 ± 2.23%, *n* = 156, *p* < 0.05; ATF7‐KD + SB202190 0.5 μM group, 29.54 ± 1.24%, *n* = 156, *p* > 0.05). Therefore, 0.1 μM SB202190 was used in further experiments. In addition, inhibition of P38 activity significantly increased the fluorescence signal intensity of HP1 and H3K9me2 in embryos in the ATF7‐KD + 0.1 μM SB202190 group compared with the ATF7‐KD group (HP1: control group, 1.00, *n* = 37; ATF7‐KD group, 0.65 ± 0.02, *n* = 34, *p* < 0.01; ATF7‐KD + SB202190 0.1 μM group, 0.98 ± 0.02, *n* = 31, *p* < 0.05; H3K9me2: control group, 1.00, *n* = 43; ATF7‐KD group, 0.72 ± 0.04, *n* = 42, *p* < 0.01; ATF7‐KD + SB202190 0.1 μM group, 0.98 ± 0.03, *n* = 37, *p* < 0.05) (Figure 4B–D). These results suggest that active P38 negatively regulates ATF7‐dependent heterochromatin function.

### 3.5 Effects of inhibition of P38 on HT embryonic development

**FIGURE 5:**
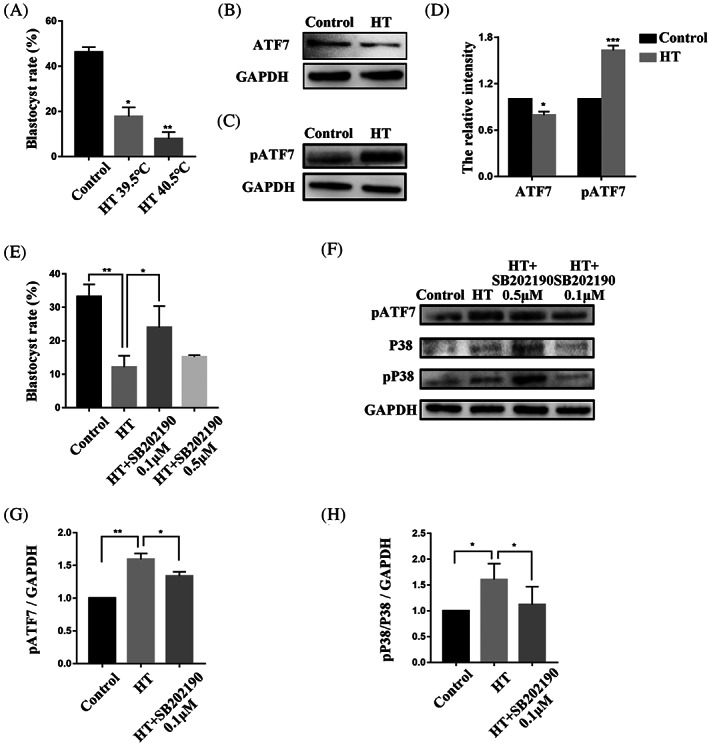
Effects of inhibition of P38 on HT embryonic development. (A) Blastocyst rate after HT exposure at 39.5 and 40.5°C. The rate decreased significantly in both HT groups compared with the control group. *p < 0.05; **p < 0.01. (B) Western blotting result of ATF7 protein expression after HT exposure in the 4C stage. (C) Western blotting result of pATF7 protein expression after HT exposure in the 4C stage. (D) Band intensity analysis of ATF7 and pATF7 after HT exposure at 39.5°C. The expression level of pATF7 protein was increased significantly in the HT group compared with the control group and the expression level of ATF7 protein was decreased significantly. *p < 0.05; ***p < 0.001 (E) Blastocyst rate after HT exposure at 39.5°C and treatment with SB202190 at 0.1 and 0.5 μM. The rate decreased significantly in the HT 39.5°C group compared with the control group. The blastocyst rate increased significantly in the HT + SB202190 0.1 μM group compared with the HT group. *p < 0.05; **p < 0.01. (F) Western blots showing pATF7, P38, and pP38 protein expression after HT exposure in the 4C stage. (G and H) Band intensity analysis of pATF7 and ratio of pP38/P38 after HT exposure at 39.5°C and treatment with SB202190 at 0.1 μM. Expression of pATF7 protein expression and the pP38/P38 ratio increased significantly in the HT group compared with the control group. The expression of pATF7 protein and the pP38/P38 ratio decreased significantly in the HT + SB202190 0.1 μM group compared with the HT group. *p < 0.05; **p < 0.01

To correlate the function of ATF7 with its stress context, we used HT treatments of 39.5 and 40.5°C to culture embryos during early porcine embryonic development. Most of the embryos in the control group developed to the blastocyst stage, while only a small proportion of the embryos in the 39.5 and 40.5°C HT groups achieved this development (control group, 46.19 ± 2.25%, *n* = 275; HT 39.5°C group, 17.75 ± 4.00%, *n* = 275, *p* < 0.05; HT 40.5°C group, 7.90 ± 2.99%, *n* = 272, *p* < 0.01) (Figure 5A). In subsequent studies, the temperature of 39.5°C was used. In addition, HT significantly induced the protein expression level of pATF7 and decreased the protein expression level of ATF7 in comparison with the control group (pATF7: control group, 1.00, *n* = 400; HT group, 1.63 ± 0.006, *n* = 400, *p* < 0.001; ATF7: control group, 1.00, *n* = 360; HT group, 0.79 ± 0.005, *n* = 360, *p* < 0.05) (Figure 5B–D). At a concentration of 0.1 μM, SB202190 significantly increased the blastocyst rate of embryos in the HT group compared with that in the control group (control group, 33.17 ± 3.70%, *n* = 418; HT group, 12.11 ± 3.41%, *n* = 479, *p* < 0.01; HT + SB202190 0.1 μM group, 23.97 ± 6.41%, *n* = 367, *p* < 0.05; HT + SB202190 0.5 μM group, 15.14 ± 0.56%, *n* = 324, *p* > 0.05) (Figure 5E). Various environmental stressors, such as heat stress, can activate P38.^20^ Our results showed that 0.1 μM SB202190 decreased the pP38/P38 ratio in the HT group and abolished the phosphorylation of ATF7 induced by HT. Densitometry analysis results (Figure 5F–H) were also consistent with these results (pATF7: control group, 1.00, *n* = 240; HT group, 1.59 ± 0.05, *n* = 240, *p* < 0.01; HT + SB202190 0.1 μM group, 1.34 ± 0.04, *n* = 240, *p* < 0.05; pP38/P38: control group, 1.00, *n* = 320; HT group, 1.60 ± 0.15, *n* = 320, *p* < 0.05; HT + SB202190 0.1 μM group, 1.12 ± 0.17, *n* = 320, *p* < 0.05). These results suggest that inhibition of P38 activity could alleviate the injury of porcine early embryos at HT.

### 3.6 Effects of inhibition of P38 on HT embryonic heterochromatin

**FIGURE 6:**
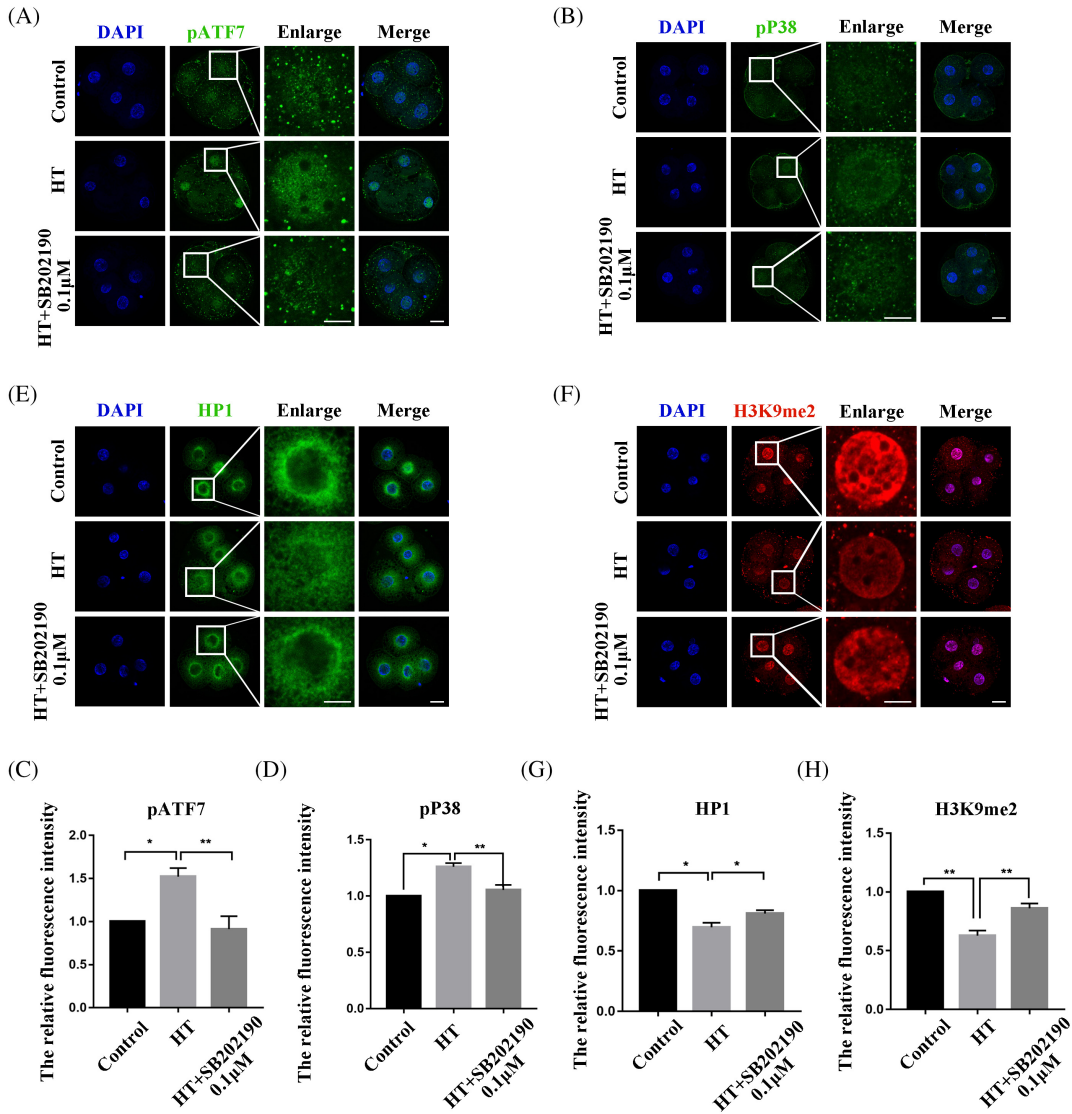
Effects of inhibition of P38 on HT embryonic heterochromatin. (A) Typical pictures of pATF7 intensity in the 4C stage after HT exposure at 39.5°C and treatment with inhibitor SB202190 at 0.1 μM. Blue, DAPI; green, pATF7. (B) Typical pictures of pP38 intensity in the 4C stage after HT exposure at 39.5°C and treatment with inhibitor SB202190 at 0.1 μM. Blue, DAPI; green, pP38. (C,D) Fluorescence intensity of pATF7 and pP38 in the 4C stage after HT exposure at 39.5°C and treatment with SB202190 at 0.1 μM. The relative fluorescence intensity of pATF7 and pP38 in the 4C stage in the HT group compared with the control group. The relative fluorescence intensity of pATF7 and pP38 were significantly higher in the HT + SB202190 0.1 μM group compared with the HT group. *p < 0.05; **p < 0.01. (E) Typical pictures of HP1 intensity in the 4C stage after HT exposure at 39.5°C and treatment with SB202190 at 0.1 μM. Blue, DAPI; green, HP1. (F) Typical pictures of H3K9me2 intensity in the 4C stage after HT exposure at 39.5°C and treatment with SB202190 at 0.1 μM. Blue, DAPI; green, H3K9me2. (G,H) Fluorescence intensity of HP1 and H3K9me2 in the 4C stage after HT exposure at 39.5°C and treatment with SB202190 at 0.1 μM. The relative fluorescence intensity of HP1 and H3K9me2 in the 4C stage in the HT group were significantly lower compared with the control group and were significantly higher in the HT + SB202190 0.1 μM group compared with the HT group. *p < 0.05; **p < 0.01

Consistent with previous results, fluorescence signal intensities of pATF7 and pP38 were significantly increased in the HT group, and these changes could be alleviated using 0.1 μM SB202190 concurrent with HT treatment (pATF7: control group, 1.00, *n* = 27; HT group, 1.52 ± 0.10, *n* = 36, *p* < 0.05; HT + SB202190 0.1 μM group, 0.91 ± 0.15, *n* = 31, *p* < 0.01; pP38: control group, 1.00, *n* = 32; HT group, 1.26 ± 0.03, *n* = 42, *p* < 0.05; HT + SB202190 0.1 μM group, 1.05 ± 0.04, *n* = 36, *p* < 0.01) (Figure 6A–D). Furthermore, we confirmed the effects of inhibiting P38 activity on the heterochromatin of embryos treated at HT. Immunofluorescence staining was used to stain the different heterochromatin marker proteins. Quantitative analysis of the fluorescence intensities of HP1 and H3K9me2 proteins in the 4C stage in the control and HT groups showed that HT significantly reduced the signal intensities of these two proteins and that these reductions could be improved in the HT + SB202190 0.1 μM group (HP1: control group, 1.00, *n* = 28; HT group, 0.70 ± 0.04, *n* = 36, *p* < 0.05; HT + SB202190 0.1 μM group, 0.81 ± 0.03, *n* = 33, *p* < 0.05; H3K9me2: control group, 1.00, *n* = 61; HT group, 0.63 ± 0.04, *n* = 53, *p* < 0.01; HT + SB202190 0.1 μM group, 0.86 ± 0.04, *n* = 45, *p* < 0.01) (Figure 6E–H).

## 4 DISCUSSION

The current study aimed to explore the role of ATF7 in early porcine embryonic development. The results demonstrate that ATF7 contributes to heterochromatin by maintaining the stable expression of H3K9me2 and HP1. In response to HT, activation of pATF7 results in the release of ATF7 together with H3K9me2, which affects the quality of early porcine embryos.

As a member of the ATF2 subfamily, ATF7 is functionally and structurally related to ATF2,^15^ which epigenetically controls gene silencing associated with heterochromatin. Heterochromatin exists in two facultative and constitutive forms. Facultative heterochromatin is a flexible form found in various chromosomal regions when the repression of gene‐coding regions is desired. Constitutive heterochromatin forms in specific regions of the genome and is characterized by arrays of tandem DNA repeats.^36^ In HeLa cells, ATF7 exhibits two localization patterns: a nuclear localization that is diffusely distributed and excludes the nucleoli, and a distinct pattern restricted to the periphery of the nucleus.^37^ In fission yeast, the homologue of ATF‐2, Atf1, plays a role in heterochromatin nucleation and interacts with Swi6, the yeast homologue of HP1.^24^ In *Drosophila* gland cells, dATF‐2 co‐precipitates with HP1. Immunostaining of polyline chromosomes revealed that both dATF‐2 and HP1 are located in heterochromatic centers.^38^ Our data indicates that ATF7 is expressed at all stages of early porcine embryonic development and enters the nucleus at the 4C stage. Subsequently, ATF7 localizes to pericentric heterochromatin, which is a type of constitutive heterochromatin. Heterochromatin maintains pericentromeric DNA and is important for normal cellular functions.^39^ This chromatin typically features accumulated inhibitory histone markers, such as H3K9me2/3,^40^ which leads to the recruitment of heterochromatin proteins and contributes to the establishment of heterochromatin and maintenance of this chromatin state.^41^, ^42^ In the present study, ATF7 co‐localized with HP1 on pericentric heterochromatin from late‐4C to BL, suggesting a potential relationship of ATF7 with heterochromatin.

To explore the potential function of ATF7 during early porcine embryonic development, we microinjected *ATF7* dsRNA to knockdown ATF7 expression in embryos. After ATF7 knockdown, blastocyst formation and quality were detrimentally affected, suggesting that ATF7 is essential for embryonic development. In addition, deletion of ATF7 resulted in decreased the H3K9me2/3 methyltransferases *G9a* and *SUV39H2* mRNA levels and affected the expression of H3K9me2 and HP1. When P38 activity was inhibited, the changes in these two proteins were alleviated. H3K9me2/3 is an epigenetic marker heterochromatin.^43^, ^44^, ^45^ G9a and SUV39H2 can directly bind H3K9me, in addition, SUV39H1/2 also interacts with members of the heterochromatin protein 1 (HP1) family, which specifically recognizes H3K9me2/3 and contributes to the transcriptional repression and proliferation of H3K9me2/3. HP1 regulates the stability of H3K9 methyltransferases and demethylases in heterochromatin tissues.^46^ Previous studies have shown that in brown preadipocytes, loss of ATF7 leads to decreased adipose tissue mass and increase in energy expenditure, as well as that ATF7 suppresses innate immunity‐related gene expression by recruiting G9a to regulate H3K9me2 levels.^47^ In *Drosophila*, dATF2 mutant cell clones in salivary glands disrupt heterochromatin formation and function by reducing HP1 and H3K9me2 signalling in a process that is negatively regulated by the P38 upstream kinase Mekk1.^38^ Together with these previous findings, our results further support the requirement of P38‐regulated ATF7 for heterochromatin function.

Cells are constantly exposed to various stresses, such as changes in osmotic pressure, oxygen, and temperature.^48^ Heat stress is one of the most prevalent environmental stresses that negatively affects embryonic development. HT induces apoptosis in mouse follicles and increases mitochondrial ROS levels in bovine oocytes, while disrupting the arrangement of organelles in porcine oocytes.^49^, ^50^, ^51^ To confirm the changes in ATF7 function in the context of stress, we used HT treatment during early porcine embryonic development. We observed that HT induced the expression of pATF7 by activating P38, thereby reducing the levels of H3K9me2 and HP1 to affect the function of heterochromatin. These changes could be alleviated by inhibition of p38 activity. In a study on mouse liver cells, a low‐protein diet induced the phosphorylation of ATF7 by P38 via ROS, which resulted in the release of ATF7 and reduction of H3K9me2 on its target genes.^52^ Similar results were also demonstrated in senescent adipocytes, where ATF7 from the promoter of the p16Ink4a gene, which encodes a cyclin‐dependent kinase inhibitor, was released as mice aged, leading to an increase in its mRNA levels. These findings suggest that ATF7 can regulate the lifespan of mice.^53^ Both osmotic stress and HT can induce dATF2 phosphorylation in *Drosophila* via the Mekk1‐p38‐dATF‐2 signalling pathway, resulting in disrupted heterochromatin. Taken together, our results demonstrate that stress‐induced heterochromatin changes during early porcine embryonic development are dependent on ATF7 under HT stress.

## 5 CONCLUSION

**FIGURE 7:**
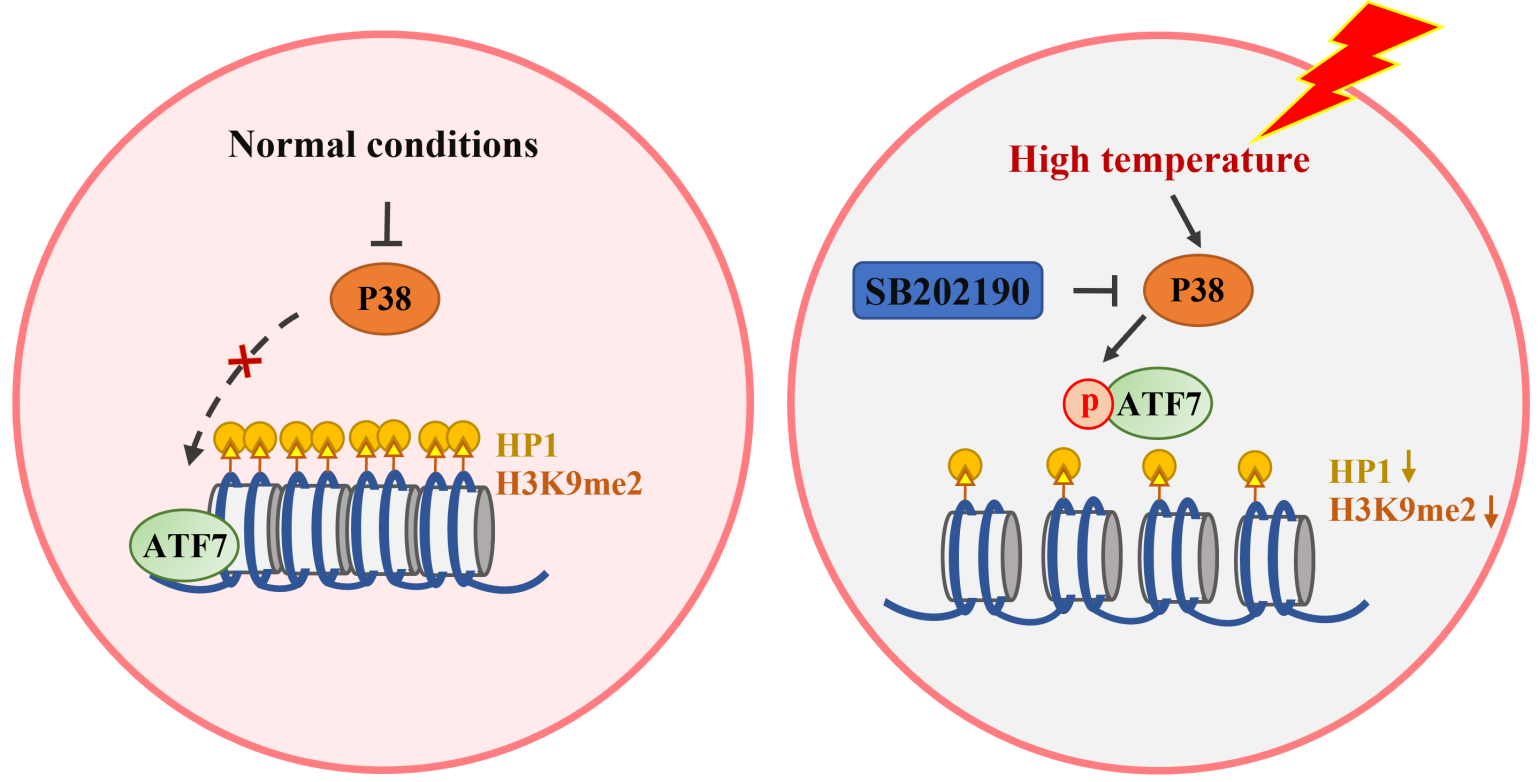
Diagram of heat‐induced ATF7‐dependent epigenetic changes during early porcine embryonic development. ATF7 plays a role in maintaining heterochromatin function. This may be through the P38‐pATF7 signalling pathway to stabilize HP1 and H3K9me2 expression during early porcine embryonic development in response to HT

ATF7 contributes to heterochromatin by maintaining the expression of H3K9me2 and HP1 in the absence of stress. In response to HT conditions, P38 is activated in the embryo. ATF7, phosphorylated by P38, is released from heterochromatin and leads to reduction of H3K9me2 and HP1 levels, ultimately adversely affecting early porcine embryonic development (Figure 7).

## AUTHOR CONTRIBUTIONS

MHS, XSC designed the experiments; MHS performed the majority of experiments; DZ, WJJ, XHL, SHL, GH, JSC, KSK, WL, XSC contributed to the materials; MHS wrote the manuscript. All authors approved the submission of the manuscript.

## FUNDING INFORMATION

This work was supported by the National Research Foundation (NRF) of Korea grant funded by the Korea Government (MSIT) (Nos. 2020R1A4A1017552 and 2022R1A2C300769), Republic of Korea.

## CONFLICT OF INTEREST

The authors declare no conflict of interests.

## INFORMED CONSENT

The authors agree with publishing this manuscript.

## Data Availability

All data generated or analysed during this study are included in this published article.

## References

[1] Liu J , Ali M , Zhou Q . Establishment and evolution of heterochromatin. Ann N Y Acad Sci. 2020;1476(1):59‐77. 32017156 10.1111/nyas.14303 PMC7586837

[2] Morrison O , Thakur J . Molecular complexes at euchromatin, heterochromatin and centromeric chromatin. Int J Mol Sci. 2021;22(13):6922. 34203193 10.3390/ijms22136922 PMC8268097

[3] Radman‐Livaja M , Rando OJ . Nucleosome positioning: how is it established, and why does it matter? Dev Biol. 2010;339(2):258‐266. 19527704 10.1016/j.ydbio.2009.06.012 PMC2830277

[4] Penagos‐Puig A , Furlan‐Magaril M . Heterochromatin as an important driver of genome organization. Front Cell Dev Biol. 2020;8:579137. 33072761 10.3389/fcell.2020.579137 PMC7530337

[5] Cabianca DS , Gasser SM . Spatial segregation of heterochromatin: uncovering functionality in a multicellular organism. Nucleus. 2016;7(3):301‐307. 27187571 10.1080/19491034.2016.1187354 PMC4991237

[6] Debey P , Renard JP , Coppey‐Moisan M , Monnot I , Geze M . Dynamics of chromatin changes in live one‐cell mouse embryos: a continuous follow‐up by fluorescence microscopy. Exp Cell Res. 1989;183(2):413‐433. 2767157 10.1016/0014-4827(89)90401-1

[7] Laurincik J , Kopecny V , Hyttel P . Detailed analysis of pronucleus development in bovine zygotes in vivo: ultrastructure and cell cycle chronology. Mol Reprod Dev. 1996;43(1):62‐69. 8720114 10.1002/(SICI)1098-2795(199601)43:1<62::AID-MRD8>3.0.CO;2-S

[8] Laurincik J , Bjerregaard B , Strejcek F , et al. Nucleolar ultrastructure and protein allocation in in vitro produced porcine embryos. Mol Reprod Dev. 2004;68(3):327‐334. 15112326 10.1002/mrd.20088

[9] Tesarik J , Kopecny V . Development of human male pronucleus: ultrastructure and timing. Gamete Res. 1989;24(2):135‐149. 2793054 10.1002/mrd.1120240203

[10] Zenk F , Zhan Y , Kos P , et al. HP1 drives de novo 3D genome reorganization in early Drosophila embryos. Nature. 2021;593(7858):289‐293. 33854237 10.1038/s41586-021-03460-z PMC8116211

[11] Sparmann A . HP1 shapes embryonic genome architecture. Nat Struct Mol Biol. 2021;28(5):412. 33972787 10.1038/s41594-021-00594-6

[12] Kageyama S , Liu H , Kaneko N , Ooga M , Nagata M , Aoki F . Alterations in epigenetic modifications during oocyte growth in mice. Reproduction. 2007;133(1):85‐94. 17244735 10.1530/REP-06-0025

[13] Liu H , Kim JM , Aoki F . Regulation of histone H3 lysine 9 methylation in oocytes and early pre‐implantation embryos. Development. 2004;131(10):2269‐2280. 15102709 10.1242/dev.01116

[14] Zylicz JJ , Borensztein M , Wong FC , et al. G9a regulates temporal preimplantation developmental program and lineage segregation in blastocyst. Elife. 2018;7:e33361. 29745895 10.7554/eLife.33361 PMC5959720

[15] Gaire M , Chatton B , Kedinger C . Isolation and characterization of two novel, closely related ATF cDNA clones from HeLa cells. Nucleic Acids Res. 1990;18(12):3467‐3473. 1694576 10.1093/nar/18.12.3467 PMC330998

[16] Hai TW , Liu F , Coukos WJ , Green MR . Transcription factor ATF cDNA clones: an extensive family of leucine zipper proteins able to selectively form DNA‐binding heterodimers. Genes Dev. 1989;3(12B):2083‐2090. 2516827 10.1101/gad.3.12b.2083

[17] Maekawa T , Sakura H , Kanei‐Ishii C , et al. Leucine zipper structure of the protein CRE‐BP1 binding to the cyclic AMP response element in brain. EMBO J. 1989;8(7):2023‐2028. 2529117 10.1002/j.1460-2075.1989.tb03610.x PMC401081

[18] Takeda J , Maekawa T , Sudo T , et al. Expression of the CRE‐BP1 transcriptional regulator binding to the cyclic AMP response element in central nervous system, regenerating liver, and human tumors. Oncogene. 1991;6(6):1009‐1014. 1829805

[19] Chang L , Karin M . Mammalian MAP kinase signalling cascades. Nature. 2001;410(6824):37‐40. 11242034 10.1038/35065000

[20] Craig CR , Fink JL , Yagi Y , Ip YT , Cagan RL . A Drosophila p38 orthologue is required for environmental stress responses. EMBO Rep. 2004;5(11):1058‐1063. 15514678 10.1038/sj.embor.7400282 PMC1299177

[21] De Graeve F , Bahr A , Sabapathy KT , et al. Role of the ATFa/JNK2 complex in Jun activation. Oncogene. 1999;18(23):3491‐3500. 10376527 10.1038/sj.onc.1202723

[22] Yoshida K , Maekawa T , Zhu Y , et al. The transcription factor ATF7 mediates lipopolysaccharide‐induced epigenetic changes in macrophages involved in innate immunological memory. Nat Immunol. 2015;16(10):1034‐1043. 26322480 10.1038/ni.3257

[23] Maekawa T , Kim S , Nakai D , et al. Social isolation stress induces ATF‐7 phosphorylation and impairs silencing of the 5‐HT 5B receptor gene. EMBO J. 2010;29(1):196‐208. 10.1038/emboj.2009.318 PMC2808367 19893493

[24] Jia S , Noma K , Grewal SI . RNAi‐independent heterochromatin nucleation by the stress‐activated ATF/CREB family proteins. Science. 2004;304(5679):1971‐1976. 15218150 10.1126/science.1099035

[25] Maekawa T , Liu B , Nakai D , et al. ATF7 mediates TNF‐alpha‐induced telomere shortening. Nucleic Acids Res. 2018;46(9):4487‐4504. 29490055 10.1093/nar/gky155 PMC5961373

[26] Yoshida K , Ishii S . Innate immune memory via ATF7‐dependent epigenetic changes. Cell Cycle. 2016;15(1):3‐4. 26556024 10.1080/15384101.2015.1112687 PMC4825762

[27] Nitta M , Yogo K , Ohashi M , et al. Identification and expression analysis of connexin‐45 and connexin‐60 as major connexins in porcine oocytes. J Anim Sci. 2010;88(10):3269‐3279. 20562362 10.2527/jas.2009-2781

[28] Tseng JK , Tang PC , Ju JC . In vitro thermal stress induces apoptosis and reduces development of porcine parthenotes. Theriogenology. 2006;66(5):1073‐1082. 16626798 10.1016/j.theriogenology.2006.03.003

[29] Edwards JL , Bogart AN , Rispoli LA , Saxton AM , Schrick FN . Developmental competence of bovine embryos from heat‐stressed ova. J Dairy Sci. 2009;92(2):563‐570. 19164666 10.3168/jds.2008-1495

[30] Roth Z , Arav A , Bor A , Zeron Y , Braw‐Tal R , Wolfenson D . Improvement of quality of oocytes collected in the autumn by enhanced removal of impaired follicles from previously heat‐stressed cows. Reproduction. 2001;122(5):737‐744. 11690534

[31] Zhou D , Niu Y , Cui X‐S . M‐RAS regulate CDH1 function in blastomere compaction during porcine embryonic development. J Anim Reprod Biotechnol. 2020;35(1):12‐20.

[32] Niu YJ , Zhou D , Cui XS . S‐nitrosoglutathione reductase maintains mitochondrial homeostasis by promoting clearance of damaged mitochondria in porcine preimplantation embryos. Cell Prolif. 2021;54(3):e12990. 33458941 10.1111/cpr.12990 PMC7941228

[33] Zhou D , Sun MH , Lee SH , Cui XS . ROMO1 is required for mitochondrial metabolism during preimplantation embryo development in pigs. Cell Div. 2021;16(1):7. 34915903 10.1186/s13008-021-00076-7 PMC8680150

[34] Niu Y‐J , Zhou D , Zhou W , et al. Nitric oxide‐induced protein S‐nitrosylation causes mitochondrial dysfunction and accelerates post‐ovulatory aging of oocytes in cattle. J Anim Reprod Biotechnol. 2020;35(1):102‐111.

[35] Zhou D , Li X‐H , Lee S‐H , Heo G , Cui X‐S . Effects of alpha‐linolenic acid and essential amino acids on the proliferation and differentiation of C2C12 myoblasts. J Anim Reprod Biotechnol. 2022;37(1):17‐26.

[36] Magaraki A , van der Heijden G , Sleddens‐Linkels E , et al. Silencing markers are retained on pericentric heterochromatin during murine primordial germ cell development. Epigenetics Chromatin. 2017;10:11. 28293300 10.1186/s13072-017-0119-3 PMC5346203

[37] Hamard PJ , Boyer‐Guittaut M , Camuzeaux B , et al. Sumoylation delays the ATF7 transcription factor subcellular localization and inhibits its transcriptional activity. Nucleic Acids Res. 2007;35(4):1134‐1144. 17264123 10.1093/nar/gkl1168 PMC1851647

[38] Seong KH , Li D , Shimizu H , Nakamura R , Ishii S . Inheritance of stress‐induced, ATF‐2‐dependent epigenetic change. Cell. 2011;145(7):1049‐1061. 21703449 10.1016/j.cell.2011.05.029

[39] Peters AH , O'Carroll D , Scherthan H , et al. Loss of the Suv39h histone methyltransferases impairs mammalian heterochromatin and genome stability. Cell. 2001;107(3):323‐337. 11701123 10.1016/s0092-8674(01)00542-6

[40] Rea S , Eisenhaber F , O'Carroll D , et al. Regulation of chromatin structure by site‐specific histone H3 methyltransferases. Nature. 2000;406(6796):593‐599. 10949293 10.1038/35020506

[41] Bannister AJ , Zegerman P , Partridge JF , et al. Selective recognition of methylated lysine 9 on histone H3 by the HP1 chromo domain. Nature. 2001;410(6824):120‐124. 11242054 10.1038/35065138

[42] Lachner M , O'Sullivan RJ , Jenuwein T . An epigenetic road map for histone lysine methylation. J Cell Sci. 2003;116(Pt 11):2117‐2124. 12730288 10.1242/jcs.00493

[43] Martens JH , O'Sullivan RJ , Braunschweig U , et al. The profile of repeat‐associated histone lysine methylation states in the mouse epigenome. EMBO J. 2005;24(4):800‐812. 15678104 10.1038/sj.emboj.7600545 PMC549616

[44] Mikkelsen TS , Ku M , Jaffe DB , et al. Genome‐wide maps of chromatin state in pluripotent and lineage‐committed cells. Nature. 2007;448(7153):553‐560. 17603471 10.1038/nature06008 PMC2921165

[45] Canzio D , Chang EY , Shankar S , et al. Chromodomain‐mediated oligomerization of HP1 suggests a nucleosome‐bridging mechanism for heterochromatin assembly. Mol Cell. 2011;41(1):67‐81. 21211724 10.1016/j.molcel.2010.12.016 PMC3752404

[46] Maeda R , Tachibana M . HP1 maintains protein stability of H3K9 methyltransferases and demethylases. EMBO Rep. 2022;23(4):e53581. 35166421 10.15252/embr.202153581 PMC8982598

[47] Liu Y , Maekawa T , Yoshida K , Muratani M , Chatton B , Ishii S . The transcription factor ATF7 controls adipocyte differentiation and thermogenic gene programming. iScience. 2019;13:98‐112. 30826729 10.1016/j.isci.2019.02.013 PMC6402263

[48] Summers MC , Biggers JD . Chemically defined media and the culture of mammalian preimplantation embryos: historical perspective and current issues. Hum Reprod Update. 2003;9(6):557‐582. 14714592 10.1093/humupd/dmg039

[49] Li J , Gao H , Tian Z , et al. Effects of chronic heat stress on granulosa cell apoptosis and follicular atresia in mouse ovary. J Anim Sci Biotechnol. 2016;7:57. 27708774 10.1186/s40104-016-0116-6 PMC5043540

[50] Payton RR , Rispoli LA , Nagle KA , et al. Mitochondrial‐related consequences of heat stress exposure during bovine oocyte maturation persist in early embryo development. J Reprod Dev. 2018;64(3):243‐251. 29553057 10.1262/jrd.2017-160 PMC6021609

[51] Lee SH , Sun MH , Zhou D , et al. High temperature disrupts organelle distribution and functions affecting meiotic maturation in porcine oocytes. Front Cell Dev Biol. 2022;10:826801. 35252192 10.3389/fcell.2022.826801 PMC8894851

[52] Yoshida K , Maekawa T , Ly NH , et al. ATF7‐dependent epigenetic changes are required for the intergenerational effect of a paternal low‐protein diet. Mol Cell. 2020;78(3):445‐58.e6. 32197065 10.1016/j.molcel.2020.02.028

[53] Maekawa T , Liu B , Liu Y , et al. Stress‐induced and ATF7‐dependent epigenetic change influences cellular senescence. Genes Cells. 2019;24(9):627‐635. 31294895 10.1111/gtc.12713

